# Long-read metagenomics reveals stable resistome and microbiome in treated Italian slaughterhouse wastewater: a preliminary study

**DOI:** 10.1128/spectrum.01562-26

**Published:** 2026-06-22

**Authors:** Stefano Pallotti, Maria Elena Nigro, Elisa Albini, Elisa Russo, Francesco M. Carpi, Maurizio Falconi, Benedetta Torbidoni-Baldassari, Anna Maria Giuliodori, Dezemona Petrelli, Livia Beccacece, Giovanni Pezzotti, Chiara Francesca Magistrali, Francesca Romana Massacci, Valerio Napolioni

**Affiliations:** 1 School of Biosciences and Veterinary Medicine, University of Camerino 18959, Camerino, Italy; 2 Istituto Zooprofilattico Sperimentale dell'Umbria e delle Marche "Togo Rosati" 34399, Perugia, Italy; 3 SYNBIOTEC Laboratori S.r.l, Camerino, Italy; 4 Istituto Zooprofilattico Sperimentale della Lombardia e dell’Emilia-Romagna "Bruno Ubertini" 18207, Brescia, Italy; University at Albany, Albany, New York, USA

**Keywords:** antimicrobial resistance, slaughterhouse wastewater, metagenomics, antibiotic resistance genes

## Abstract

Antimicrobial resistance (AMR) poses a major threat to global health, and food production environments are increasingly recognized as potential reservoirs and dissemination points for resistant bacteria and antimicrobial resistance genes (ARGs). Slaughterhouse wastewater contains complex microbial communities originating from multiple animal sources and processing activities, yet the effectiveness of current treatment processes in mitigating microbiological and resistome-associated risks remains poorly understood. In this study, we applied high-throughput long-read metagenomic sequencing to characterize microbial community composition and resistome profiles in wastewater samples collected before and after physicochemical treatment from four Italian slaughterhouses. Taxonomic profiling revealed a diverse microbiome dominated by *Bacillota* and *Pseudomonadota*, along with DNA assigned to potentially clinically relevant taxa, including members of the ESKAPE group. Resistome analysis identified 96 ARGs conferring resistance to 16 antimicrobial classes. Comparative analyses of pre- and post-treatment samples showed no significant changes in microbial community structure, alpha- and beta-diversity metrics, or ARG profiles. These findings indicate that the applied coagulation–flocculation-based treatment has limited effects on the relative composition of the wastewater microbiome and resistome, as detected by shotgun metagenomics. Our results suggest that slaughterhouse wastewater may act as a persistent environmental reservoir of antimicrobial resistance determinants and highlight the need for enhanced treatment strategies and resistome-oriented surveillance within a One Health framework. Given the limited sample size and the preliminary nature of this investigation, these findings should be interpreted as exploratory and hypothesis-generating, rather than broadly generalizable.

**IMPORTANCE:**

Antimicrobial resistance is a growing global health concern that extends beyond clinical settings into agricultural and environmental systems. Slaughterhouses represent critical interfaces where microbial communities from livestock, processing environments, and wastewater converge, creating opportunities for the persistence and dissemination of antimicrobial resistance genes. Despite the widespread use of physicochemical treatments to reduce organic load and suspended solids in slaughterhouse wastewater, their impact on microbial communities and resistome remains poorly characterized. By applying long-read metagenomic sequencing, this study provides a comprehensive characterization of the microbiome and resistome in slaughterhouse wastewater before and after treatment. Our findings show that commonly applied coagulation–flocculation treatments do not substantially alter the relative structure of microbial communities or the diversity of resistance genes. These results highlight the potential role of slaughterhouse wastewater as an environmental reservoir for antimicrobial resistance and emphasize the need for improved treatment technologies and systematic surveillance strategies to mitigate the environmental dissemination of resistance determinants in line with the One Health approach.

## INTRODUCTION

Antimicrobial resistance (AMR) is one of the most pressing global health challenges, jeopardizing effective treatment of infectious diseases and increasing morbidity and mortality worldwide (1, 2). In 2019 alone, bacterial AMR was estimated to be directly responsible for over 1.27 million deaths and associated with nearly 5 million deaths globally, with pathogens, such as *Escherichia coli*, *Staphylococcus aureus*, *Klebsiella pneumoniae*, *Streptococcus pneumoniae*, *Acinetobacter baumannii*, and *Pseudomonas aeruginosa*, accounting for most fatalities (2). Beyond public health, the economic burden of AMR is substantial, with projected additional healthcare costs and gross domestic product losses reaching trillions of dollars globally by 2030–2050 (3).

AMR arises when microorganisms acquire the ability to withstand antimicrobial agents, including antibiotics (4). The evolution of resistance is driven by both random genetic mutations and horizontal gene transfer—the movement of genetic material between organisms other than by descent, which enables bacteria to spread antibiotic resistance genes (ARGs) across microbial populations, thereby increasing adaptability and pathogenic potential (5, 6).

The food production chain, and in particular slaughterhouses, has been recognized as a critical interface for the spread of antimicrobial-resistant bacteria and ARGs from livestock to humans and the environment (7). Carcasses and processing environments are exposed to diverse microbial populations from multiple sources, including animal hides, gastrointestinal contents, processing equipment, and personnel, creating opportunities for microbial exchange and cross-contamination (8, 9). Slaughterhouse environments are further complicated by the periodic application of disinfectants, which, while intended to mitigate microbial contamination, may co-select for bacteria harboring both ARGs and disinfectant resistance (10, 11).

Occupational exposure to AMR bacteria has also been documented among agricultural and food-industry workers, including farmers, veterinarians, and slaughterhouse staff, emphasizing the potential for occupational dissemination of ARGs from animals to humans (12).

The microbial burden in slaughterhouses and associated resistome is not limited to pathogens. Commensal bacteria can also act as persistent reservoirs of ARGs, evading routine interventions such as heat, pH adjustment, or chemical disinfection (13). This underscores the need for targeted approaches to capture low-abundance ARGs and better evaluate the effectiveness of wastewater treatment procedures.

Given the complexity of microbial dynamics in slaughterhouses, the interconnectedness between environmental and occupational exposures, and the limitations of conventional surveillance, a holistic evaluation of the resistome across processing environments is essential. Recent advances in high-throughput metagenomics have enabled a more comprehensive characterization of resistomes and microbiomes in both human- and animal-associated environments (7, 14). Metagenomic approaches provide the sensitivity required to detect low-abundance ARGs in samples with high host DNA content and offer a more accurate depiction of ARG dynamics along the food production chain. Such approaches can inform risk assessment, guide disinfection strategies, and support interventions to reduce the transmission of AMR from animals to humans and into the broader environment.

In the slaughterhouses included in this study, wastewater undergoes chemical treatment steps primarily to remove suspended solids and reduce the organic load prior to discharge. These steps typically rely on coagulation and flocculation, which promote aggregation and sedimentation of the matter. Although coagulation–flocculation is not a primary disinfection strategy, it may affect the microbial and resistome profiles of the treated wastewater by selectively removing particle-associated microorganisms and extracellular DNA. Therefore, assessing whether these treatments are associated with detectable shifts in the metagenomic composition and in the abundance of antimicrobial resistance genes is relevant for understanding the potential dissemination of resistance determinants in downstream environments.

Through integrated metagenomic and resistance profiling analyses, this work provides information to develop targeted strategies to mitigate the persistence and dissemination of antimicrobial resistance, thereby improving food safety and public health.

## RESULTS

### Quality control, sequencing, and read distribution

Sequencing of the enriched DNA libraries yielded an average of 367,980 reads per sample (range: 145,557–722,575) (Table S1A). The number of host-derived reads varied among samples; the highest numbers were observed for swine (average 28,436 reads, range: 3,072–77,232), followed by human (average 7,562 reads, range: 957–43,223), rabbit (average 750 reads, range: 13–2,141), chicken (average 47 reads, range: 10–112), sheep (average 30 reads, range: 6–53), cattle (average 14 reads, range: 8–24), and goat (average 8 reads, range: 0–17). On average, 36,849 reads mapped to different host genomes, representing approximately 11.4% of the total, whereas 331,130 reads remained unmapped, corresponding to approximately 88.59%.

All the rarefaction curves reached saturation (Fig. S1), indicating that sequencing depth was sufficient to capture most microbial diversity and that sequencing effort was adequate for downstream comparative analyses.

### Microbial community composition

**Fig 1:**
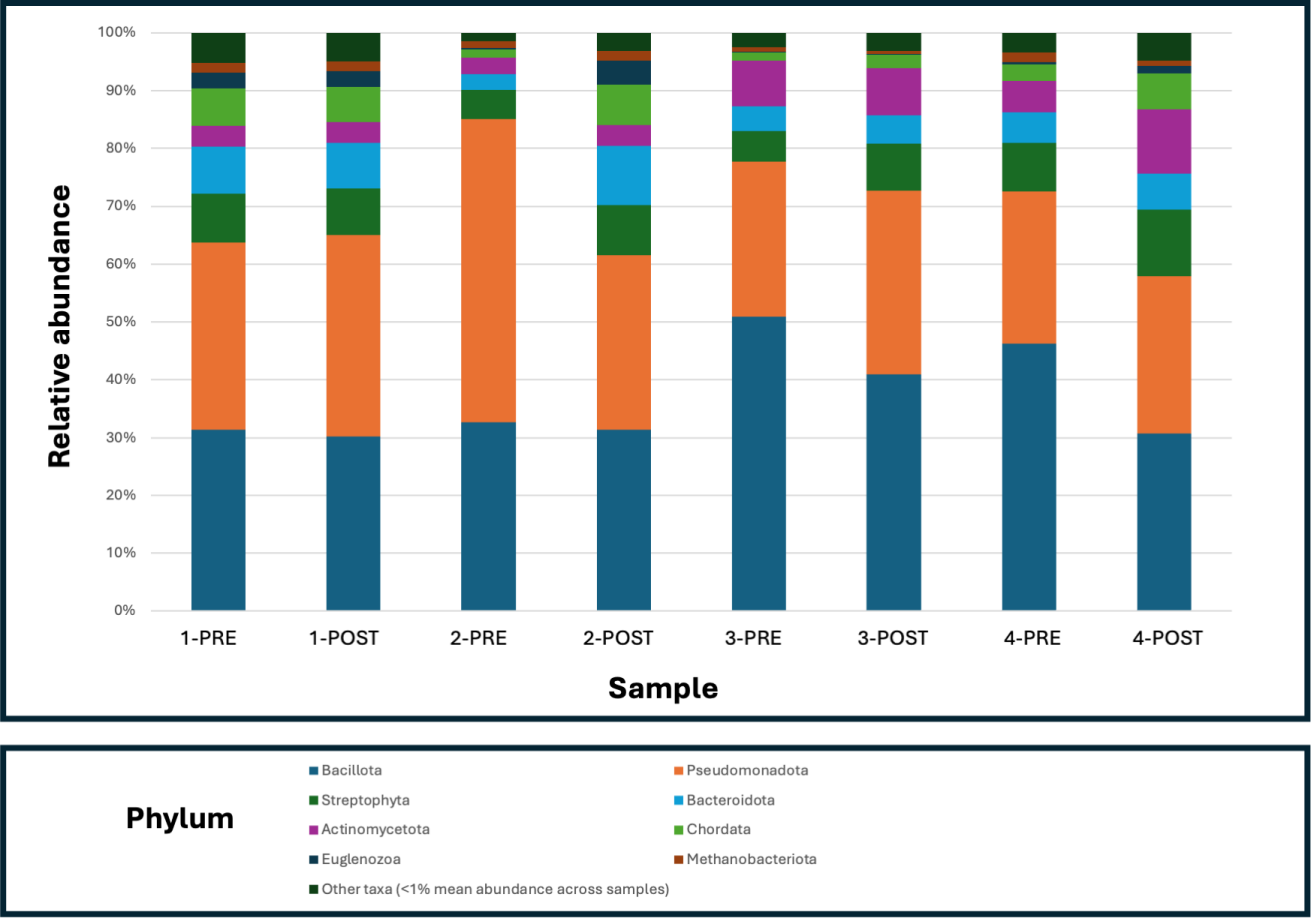
Microbial composition of wastewater samples. Stacked bar plots show the community composition of wastewater before (-PRE) and after (-POST) treatment at the phylum level.

A total of 44 phyla were identified across all wastewater samples, with *Bacillota* and *Pseudomonadota* the most abundant in pre- and post-treatment samples (Fig. 1), accounting for 30.26%–50.98% and 26.28%–52.46% of the identified phyla, respectively (Table S2). Despite observable differences in relative abundance patterns between pre- and post-treatment samples, ANCOM-BC2 analysis did not identify any taxa with statistically significant differential abundance at either the phylum or species level after correction for multiple testing (Table S3A and B). This comparison evaluated PRE versus POST conditions across all samples, accounting for the slaughterhouse as a random effect.

### Alpha- and beta-diversity and principal coordinates analysis

Alpha-diversity was assessed using the Shannon, Berger-Parker, Simpson, and Fisher indices. No significant differences were observed in any of the metrics between pre- and post-treatment samples, indicating that treatment did not alter within-sample richness and evenness (Table 1).

**TABLE 1:** Alpha diversity values were assessed using the Shannon, Berger-Parker, Simpson, and Fisher indices to evaluate richness and evenness within and between wastewater samples before and after treatment

Wastewater sample	Shannon’s diversity	Berger-Parker’s diversity	Simpson’s index of diversity	Simpson’s reciprocal index	Fisher’s index
1-PRE	5.160	0.143	0.968	31.005	135.661
1-POST	5.157	0.142	0.968	31.433	136.521
2-PRE	4.642	0.168	0.947	18.749	178.413
2-POST	5.114	0.087	0.976	41.840	106.993
3-PRE	4.845	0.288	0.913	11.530	168.576
3-POST	5.225	0.129	0.975	39.479	99.877
4-PRE	5.461	0.166	0.967	30.132	197.463
4-POST	4.931	0.146	0.965	28.869	83.685
Wilcoxon test *P* values	1	0.068	0.273	0.273	0.144

Similarly, beta-diversity and PERMANOVA analyses showed no significant differences (Tables S4A through C), suggesting that the treatment did not significantly alter the taxonomic composition of the microbial community.

**Fig 2:**
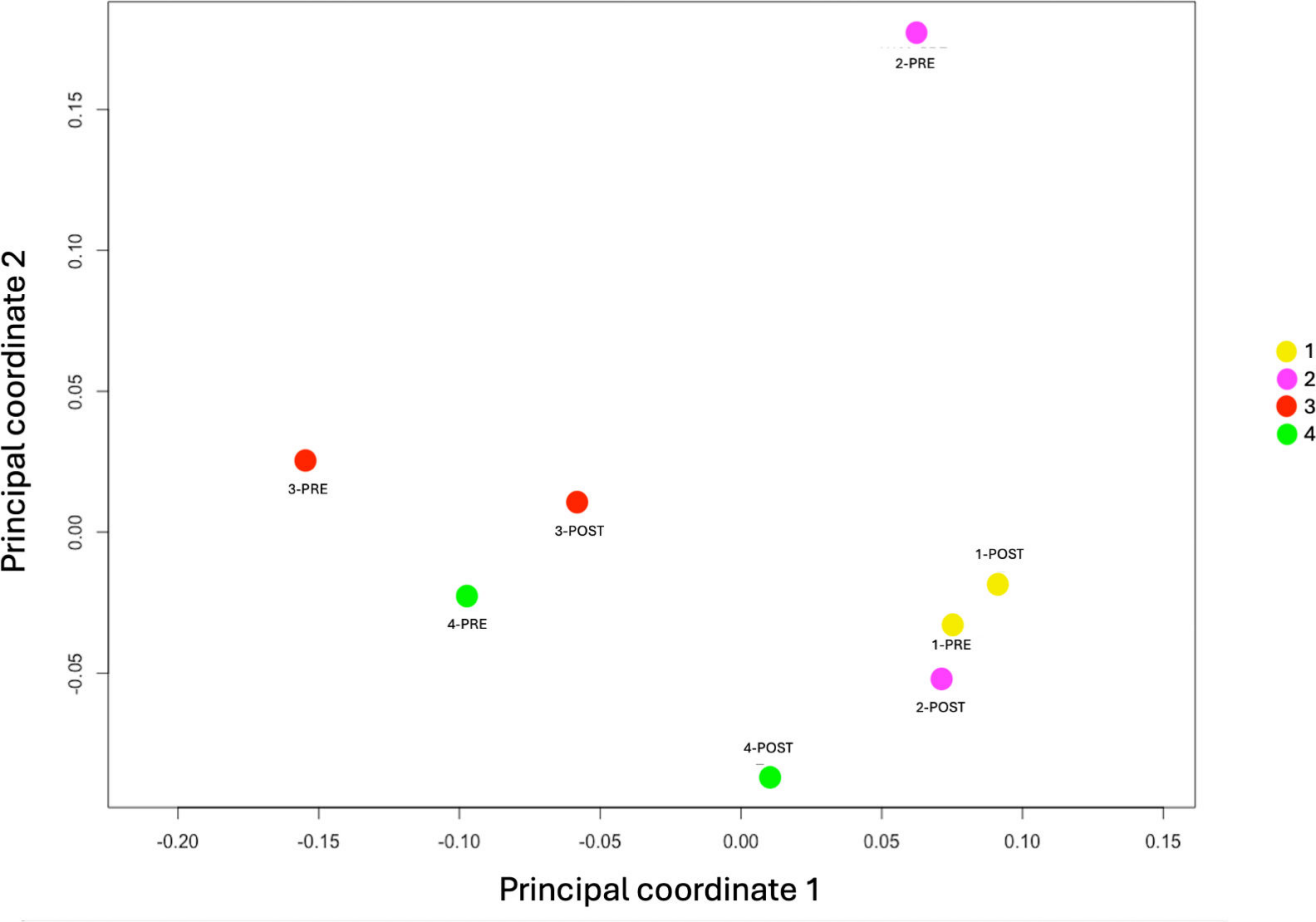
Principal coordinates analysis. Analyses were performed using the Bray–Curtis metric in the vegan (v2.5-6) package to show the dissimilarities in microbial composition across samples before (-PRE) and after (-POST) treatment.

Consistently, principal coordinates analysis (PCoA) based on Bray-Curtis dissimilarity did not reveal significant separation in microbial taxonomic composition between pre- and post-treatment wastewater samples (Fig. 2). Slight clustering was observed for samples 1-PRE/POST and 3-PRE/POST, which may reflect their shared geographical origins.

### Bacterial pathogens and ARGs analyses

Taxonomic profiling revealed sequences matching potentially clinically relevant taxa. DNA potentially belonging to ESKAPE bacterial pathogens (8) in all samples (Table S5). It is important to note that these results refer to metagenomic reads assigned to ESKAPE-associated taxa and do not imply the presence of viable pathogens, strain-level identification, or direct clinical risk. The Wilcoxon test indicated no significant differences between pre- and post-treatment samples, although a slight reduction in these bacteria was observed in the “3-POST” sample.

Moreover, ResFinder identified 96 ARG across all samples (Table S6A), conferring resistance to drugs from 16 antimicrobial classes (Table S6B). Detected ARGs showed high sequence similarity to reference sequences. Mean coverage was 95.5% (SD 6.4), while mean identity was 96.7% (SD 4.1). Median coverage and identity were 99.3% (IQR 92.7–99.8) and 98.2% (IQR 96.4–99.0), respectively. However, the Wilcoxon test indicated no significant differences between pre- and post-treatment samples; no AMR gene, whether considered individually or grouped by phenotypic resistance class, showed a significant difference between the two groups.

## DISCUSSION

This study investigates the role of slaughterhouses as critical points for the dissemination of antibiotic-resistant bacteria by applying high-resolution metagenomic approaches to assess the resistome, microbiome composition, and presence of reads assigned to DNA from potentially clinically relevant ESKAPE pathogens in wastewater before and after treatment. These assignments refer exclusively to sequence similarity-based taxonomic classification and should not be interpreted as confirmation of viable ESKAPE pathogens or strain-level identification. By integrating these analyses, we provide a comprehensive evaluation of the effectiveness of slaughterhouse hygiene interventions and their implications for the spread of antimicrobial resistance.

The sequencing strategy, based on Oxford Nanopore technology, generated a high number of reads per sample, with a large proportion of reads not mapping to host genomes (ranging from 65% to 97%). This observation reflects the complex, heterogeneous microbial communities associated with slaughterhouse environments and may also indicate the presence of poorly characterized or currently underrepresented taxa in reference databases, a known limitation of metagenomic analyses in environmental and food-related matrices (15).

In our samples, the most abundant phyla were *Bacillota* and *Pseudomonadota*, both of which are prevalent in livestock and associated environments. High abundances of these phyla have been observed in swine carcasses (16), particularly in the stomach and jejunum of pigs (17), as well as in the cecal microbiota of chickens (18). *Bacillota* has also been identified as a potential reservoir of antimicrobial-resistant bacteria dispersed within pig farms and their surrounding environments (19). Both *Bacillota* and *Pseudomonadota* appear to contribute to the dissemination of antibiotic resistance genes in aquatic ecosystems receiving poultry slaughterhouse wastewater (20). Within *Pseudomonadota*, members of the *Enterobacteriaceae* are recognized as primary hosts of antibiotic resistance genes, shaping the resistome through plasmid-mediated horizontal gene transfer across the pork production chain (21). A high prevalence of *Pseudomonadota* has also been reported in bovine and goat/sheep slaughterhouses (22). Similarly, in fecal samples from dairy cattle, the *Bacillota* phylum is the predominant, followed by *Pseudomonadota* (23). The dominance of *Bacillota* and *Pseudomonadota* in our samples suggests that slaughterhouse environments act as convergence points for microbial communities originating from multiple anatomical sites and animal species, thereby increasing opportunities for microbial exchange and resistome enrichment. The metabolic versatility, environmental resilience, and stress tolerance of many taxa within these phyla likely facilitate their persistence on carcasses and processing surfaces, promoting the maintenance and spread of antimicrobial resistance determinants throughout the slaughtering process. Their consistent detection across diverse livestock production systems further supports their potential use for monitoring microbial contamination and antimicrobial resistance dynamics in meat production environments.

However, despite these compositional patterns observed in our study, ANCOM-BC2 analysis did not identify any statistically significant differences in taxonomic abundance between pre- and post-treatment samples at either the phylum or species level after correction for multiple testing. This lack of statistical significance may be attributable, at least in part, to the limited sample size, which reduces statistical power and increases inter-site variability in compositional analyses.

Alpha-diversity, assessed using the Shannon, Berger-Parker, Simpson, and Fisher indices, varied across samples but showed no statistically significant differences between pre- and post-treatment groups.

Similarly, PERMANOVA on beta-diversity revealed no statistically significant differences between pre- and post-treatment groups, and principal coordinates analysis (PCoA) with Bray-Curtis distances showed no distinct clustering among groups. Taken together, these results indicate that the applied treatment measures have a limited impact on reshaping the global microbial community in slaughterhouse wastewater.

Analyses revealed that the most abundant bacterial DNA assigned to potentially pathogenic taxa were *Klebsiella pneumoniae* (samples 1_PRE and POST, 2_PRE), *Acinetobacter baumannii* (samples 2_POST, 3_PRE and POST, 4_PRE), and *Pseudomonas aeruginosa* (sample 4_POST). Consistent with our findings, ESKAPE bacteria have also been reported in wastewater and process water from German poultry slaughterhouses (24). Similarly, pig slaughterhouses have been shown to generate wastewater containing high concentrations of clinically relevant ESKAPE bacteria and associated antibiotic resistance genes (25). It is important to emphasize that ESKAPE pathogens may also act as zoonotic agents (26, 27) and are clinically important in hospital settings, known for their capacity to develop multiple resistances (28). It should be stressed, however, that our analyses identified long reads matching bacterial genomes potentially attributable to the ESKAPE group; however, further investigations are required to confirm the actual presence of ESKAPE bacteria in our samples. These results represent taxonomic assignments based on sequence similarity and do not provide evidence of pathogen viability, infectivity, or clinical risk.

The fact that the metagenomic profile does not change after the flocculation and coagulation procedure does not allow conclusions about microbial viability or absolute microbial load, but suggests that this treatment likely does not produce a relative enrichment or reduction of pathogenic bacteria (and/or their DNA) within the microbiome. In other words, the treatment may reduce the organic component and, consequently, the absolute number of bacteria; however, our analysis appears to show that it does not alter the relative proportions of DNA among different species, including both potentially pathogenic and non-pathogenic. This is far from negligible within the One Health approach, as increasing evidence indicates that bacteria can acquire genes through horizontal gene transfer mediated by mobile genetic elements, genes that may subsequently confer selective advantages under specific environmental conditions. In line with this, we detected multiple resistance-associated genes using ResFinder and ABRicate. Therefore, the flocculation- and coagulation-based treatments do not appear to affect the reshaping of the resistome. The same result was obtained even when the analysis was performed by grouping genes into resistance classes.

A detailed analysis of individual genes revealed that *tet(W*), associated with resistance to doxycycline, tetracycline, and minocycline (samples 1_PRE, 4_PRE, and POST), *lnu(A*), conferring lincomycin resistance (samples 1_POST and 2_PRE and POST), and *lnu(C),* also linked to lincomycin resistance (sample 3_PRE and POST), were among the most abundant antimicrobial resistance genes in our samples. Overall, the most prevalent ARGs detected across samples were those conferring resistance to aminoglycosides (1_PRE and 3_POST), lincosamides (11186_PRE and POST, 1_POST, and 3_PRE), and tetracyclines (4_PRE and POST). Similar findings were reported by Gaire (13) in a study conducted in a pig slaughterhouse, where these resistance determinants were frequently detected. The prevalence of ARGs associated with aminoglycoside and tetracycline resistance has also been documented in commercial pig slaughterhouses (12, 29).

From a public health perspective, the continued detection of ARGs conferring resistance to critically important antimicrobial classes underscores the urgent need for improved control strategies and resistome-focused monitoring in slaughterhouses.

### Conclusions

Overall, the results indicate that current treatment procedures are not effective in shifting the relative microbial profile, decreasing the presence of the DNA assigned to pathogenic bacteria (including potentially ESKAPE pathogens), and reshaping the resistome. In light of these findings, the following actions are recommended:( i) review and strengthen post-slaughter treatment; (ii) implement advanced decontamination technologies, potentially integrated with targeted approaches (e.g., selective biocides, physical or biological treatments); (iii) conduct systematic metagenomic monitoring across other sites in the production chain to track the transmission of resistant bacteria; and (iv) obtain microbial data to support the metagenomic approach.

It should be emphasized that the detection of ESKAPE-associated taxa is based on metagenomic sequence similarity and does not reflect the presence of viable or clinically active pathogens, but rather on environmental DNA signatures.

Moreover, given the limited sample size and the preliminary design of this study, these conclusions should be interpreted with caution and further validated through larger-scale investigations.

This study provides an important contribution to understanding slaughterhouses as potential environmental “hotspots” for the spread of antibiotic resistance and lays the groundwork for developing more effective mitigation strategies.

## MATERIALS AND METHODS

### Wastewater sampling and treatment

Raw sewage (pre-) and treated wastewater (post-) samples were collected from four slaughterhouses in Central Italy. Pre-treatment samples correspond to raw influent wastewater prior to on-site physicochemical treatment, whereas post-treatment samples correspond to effluent wastewater after treatment. This study was conducted from June 2023 to March 2024. The selected slaughterhouses have a capacity of 2,500 pigs per day and operate 4 days per week, with all animals sourced from national farms. Overall, eight wastewater samples were collected, comprising four pre-treatment and four post-treatment samples (one paired set per slaughterhouse). Detailed information on sampling dates and treatment stages is reported in Table S1B.

At each slaughterhouse, wastewater underwent on-site physicochemical pretreatment before discharge to the municipal wastewater treatment plant. The treatment line consisted of coarse (6 mm) and fine (1 mm) screening, followed by aeration and fine grit removal. Subsequently, wastewater was subjected to chemical coagulation–flocculation with ferric chloride and an anionic polyelectrolyte, with pH adjusted with sodium hydroxide as needed, and then treated with dissolved air flotation to remove suspended solids and associated contaminants. The clarified effluent was finally collected in an aerated holding tank before discharge to the municipal sewer system.

Pre- and post-treatment slaughterhouse wastewater samples were collected in triplicate using sterile 50-mL Falcon tubes. Triplicate samples were collected on the same day within a 1-h time window at both the influent and effluent of the physicochemical treatment line. Samples were either processed immediately or stored at −20°C until analysis.

### Wastewater sample processing: DNA extraction, library preparation, and metagenomic sequencing

These replicates were pooled prior to DNA extraction and subsequent metagenomic library preparation to obtain a representative sample for each sampling point. Genomic DNA (gDNA) was extracted from each sample using the E.Z.N.A. kit (Omega Bio-tek Inc., Norcross, GA, USA) and subsequently quantified with a Qubit 4.0 Fluorometer using the 1X dsDNA High Sensitivity (HS) assay kit. Sequencing libraries for each individual sample were prepared from 400 ng of gDNA using the Native Barcoding Kit 24 V14 (SQK-NBD114.24; Protocol ID: NBE_9169_v114_revU_30Jan2025) from Oxford Nanopore Technologies. Quality-control measurements using the Qubit were performed according to the library preparation protocol. Barcoded samples were pooled “as is,” rather than following the equimolar pooling method specified in the protocol.

Sequencing was performed on a single FLO-MIN114 (R10.4.1) flow cell using a MinION Mk1D, in accordance with the manufacturer’s instructions. The run underwent real-time base calling and demultiplexing with Dorado v7.6.7 (Super Accurate configuration), integrated into MinKNOW software (Oxford Nanopore Technologies). Only reads passing the default quality threshold (minimum *Q*-score = 10) were retained for downstream analyses. No additional minimum read-length filtering was applied.

### Bioinformatics and statistical analysis

The reads in the FASTQ files were taxonomically classified using with Kraken2 v2.1.4 (30) using default parameters against the reference genomes of the following species, in order to remove potential sample contamination due to the presence of host genomes: *Homo sapiens* (GRCh38.p14), *Sus scrofa* (Sscrofa11.1), *Oryctolagus cuniculus* (mOryCun1.1), *Gallus gallus* (bGalGal1.mat.broiler.GRCg7b), *Bos taurus* (ARS-UCD2.0), *Ovis aries* (ARS-UI_Ramb_v3.0), and *Capra hircus* (ARS1.2) (Table S1A). Reads classified as host-derived were removed, while unclassified reads were retained for downstream analyzes.

Taxonomic classification of unaligned reads was performed using Kraken2 v2.1.4 with default parameters against the PlusPFP database (accessed on 12/28/2024), which includes standard RefSeq genomes of protozoa, fungi, and plants. Rarefaction curves were generated using MicrobiomeAnalyst v2.0 (31) with default parameters to evaluate sequencing depth across samples (Fig. S1).

The relative abundance of bacterial species for each sample, both before and after treatment, was estimated with Bracken v2.9 (32) using default parameters. The sequencing data consisted of long Nanopore reads with an N50 of approximately 1 kb, reflecting a heterogeneous read-length distribution in the output. It should be noted that Bracken was originally developed for short-read sequencing data, and its application to long-read Nanopore data sets may introduce approximation biases in abundance estimation.

Alpha- and beta-diversity metrics were computed with KrakenTools (33) using default settings.

Differences in the relative abundance of individual microbial taxa between pre- and post-treatment groups were assessed using ANCOM-BC2 (34) implemented in R. Analyses were conducted at both the phylum and species levels.

At the species level, taxa were derived from Bracken outputs and filtered to include only those present in at least 10% of samples. At the phylum level, taxa were retained if present at a minimum abundance threshold (>5 counts in at least two samples) to reduce noise from extremely rare taxa. Sample metadata included treatment group (pre vs post) as a fixed effect and slaughterhouse of origin as a random effect to account for paired sampling structure. Phyloseq objects (phyloseq v1.42.0) (29) were constructed for each analysis, and ANCOM-BC2 was run with the following specifications: fixed effect = treatment, random effect = slaughterhouse, multiple testing correction = Benjamini–Hochberg false discovery rate, and significance threshold α = 0.05. Data processing and filtering were performed using tidyverse v2.0.0 packages (35).

For visualization purposes, taxonomic data were aggregated at the phylum level (Fig. 1). Given the high number of low-abundance taxa and to improve interpretability, only phyla with a mean relative abundance ≥1% across all samples were retained for graphical representation. All remaining taxa were grouped into a single category (“Other (<1%)”) by summing their relative abundances within each sample.

Importantly, the taxonomic profiling was not restricted to bacterial sequences. Consequently, certain eukaryotic taxa (e.g., *Chordata* and *Euglenozoa*) were detected and retained when exceeding the selected abundance threshold. These taxa are reported to reflect the broad-spectrum nature of the metagenomic approach and the complexity of wastewater-derived genetic material.

The resulting filtered data set was used to generate stacked bar plots showing the relative abundance of the dominant phyla and the aggregated “Other” category across all samples.

Statistical comparisons of alpha diversity metrics between PRE and POST samples were performed using the Wilcoxon test in SPSS, while differences in beta diversity between treatment groups were assessed using PERMANOVA implemented via the adonis2 function in the Vegan package v2.5-6 (36) in R (37), based on Bray–Curtis dissimilarity measure.

Principal coordinates analysis (PCoA) was performed with the Bray–Curtis dissimilarity measure using the Vegan package v2.5-6 in R.

ARGs were identified using the Epi2Me workflow (Oxford Nanopore Technologies), which employs ABRicate (v1.0.1-epi2me) (38) for screening against the ResFinder database (accessed on 30/05/2025) (39). Default detection thresholds for sequence identity and coverage were applied, and only high-confidence hits were retained. Minimum alignment length was implicitly defined by the alignment algorithm. Only acquired ARGs were retained, whereas point mutation-mediated resistance (SNP-based) was not assessed.

## Data Availability

The data associated with this research are available from the NCBI SRA under accession number PRJNA1417753.

## SUPPLEMENTAL MATERIAL

Figure S1 spectrum.01562-26-s0001.pdf Rarefaction curves showing sequencing depth and observed taxonomic richness across the eight PRE- and POST-treatment wastewater samples.

Table S1 spectrum.01562-26-s0002.docx Read alignment results and paired design of wastewater samples collected before and after treatment across slaughterhouses.

Table S2 spectrum.01562-26-s0003.xlsx Relative abundance of bacterial species for each sample, before and after treatment, at the phylum level.

Table S3 spectrum.01562-26-s0004.xlsx Differential abundance analysis at the phylum and species levels between pre- and post-treatment wastewater samples, determined using ANCOM-BC2.

Table S4 spectrum.01562-26-s0005.docx Pairwise Bray-Curtis dissimilarity values among wastewater samples before and after treatment; PERMANOVA and beta-dispersion analyses based on Bray-Curtis beta diversity comparing PRE and POST wastewater samples.

Table S5 spectrum.01562-26-s0006.xlsx ANCOM-BC2 results for bacterial taxa assigned to ESKAPE-associated groups (PRE vs POST comparison).

Table S6 spectrum.01562-26-s0007.xlsx AMR genes identified using the ResFinder software (Epi2Me platform) and the ABRicate reference database, and analysis of AMR genes grouped by antibiotic resistance class.

ASM does not own the copyrights to Supplemental Material that may be linked to, or accessed through, an article. The authors have granted ASM a non-exclusive, world-wide license to publish the Supplemental Material files. Please contact the corresponding author directly for reuse.
