# Supplementary material for: Long-read metagenomics reveals stable resistome and microbiome in treated Italian slaughterhouse wastewater: a preliminary study
Source: Microbiol Spectr. 2026 Jun 22;14(8):e01562-26. doi: 10.1128/spectrum.01562-26 (PMC13435776; doi:10.1128/spectrum.01562-26)
Supplement: Supplementary file 1 [file spectrum.01562-26-s0001.pdf]

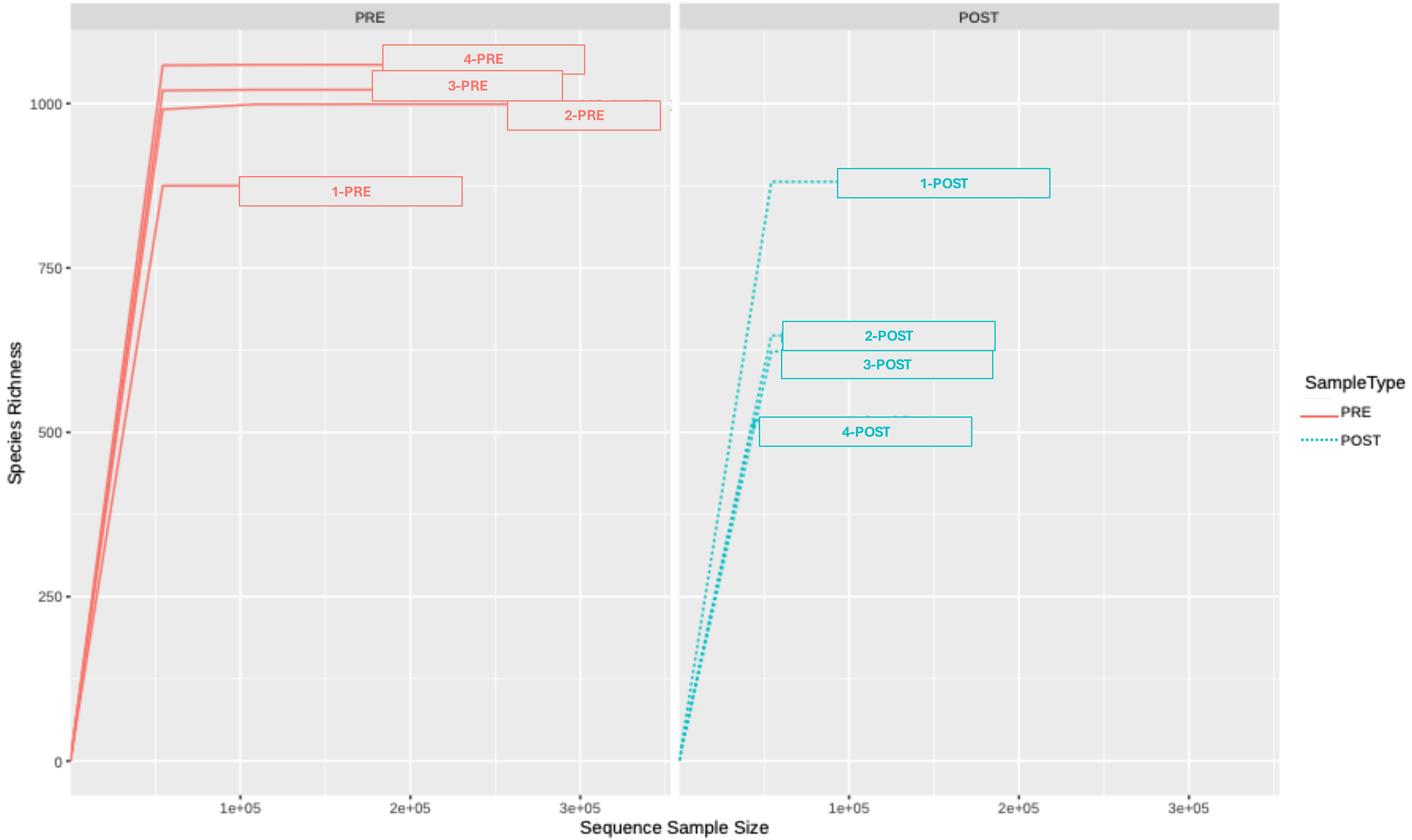
PRE
POST
4-PRE
3-PRE
2-PRE
1-PRE
1-POST
2-POST
3-POST
4-POST
Species Richness
1000
750
500
250
0
1e+05
2e+05
3e+05
Sequence Sample Size
SampleType
PRE
POST
