# Supplementary material for: Long-read metagenomics reveals stable resistome and microbiome in treated Italian slaughterhouse wastewater: a preliminary study
Source: Microbiol Spectr. 2026 Jun 22;14(8):e01562-26. doi: 10.1128/spectrum.01562-26 (PMC13435776; doi:10.1128/spectrum.01562-26)
Supplement: Supplementary file 2 [file spectrum.01562-26-s0002.docx]

**Supplementary Table 1 A. Reads alignment results**

| **Sample Name** | **1-PRE** | **1-POST** | **2-PRE** | **2-POST** | **3-PRE** | **3-POST** | **4-PRE** | **4-POST** | **Mean ± SD of reads in the eight samples** | | |
| --- | --- | --- | --- | --- | --- | --- | --- | --- | --- | --- | --- |
| **Total reads yielded by sequencing** | 722,575 | 339,285 | 530,502 | 234,391 | 348,479 | 145,557 | 471,390 | 151,662 | | 367,980,125 | 199341.711 |
| ***n* of reads mapping human genome** | 43,223 | 3,862 | 3,275 | 2,826 | 1,845 | 957 | 3,260 | 1,254 | | 7,562,750 | 14,446,023 |
|  |  |  |  |  |  |  |  |  | |  |  |
| ***n* of reads mapping swine genome** | 11,189 | 71,112 | 17,734 | 77,232 | 6,594 | 3,072 | 27,267 | 13,288 | | 28,436,000 | 29192.749 |
|  |  |  |  |  |  |  |  |  | |  |  |
| ***n* of reads mapping rabbit genome** | 520 | 1,880 | 91 | 36 | 53 | 13 | 2,141 | 1,266 | | 750,000 | 886.727 |
|  |  |  |  |  |  |  |  |  | |  |  |
| ***n* of reads mapping chicken genome** | 68 | 10 | 35 | 112 | 42 | 17 | 50 | 44 | | 47,250 | 31.896 |
|  |  |  |  |  |  |  |  |  | |  |  |
| ***n* of reads mapping sheep genome** | 44 | 37 | 53 | 21 | 29 | 6 | 38 | 17 | | 30,625 | 15.408 |
|  |  |  |  |  |  |  |  |  | |  |  |
| ***n* of reads mapping cattle genome** | 24 | 21 | 15 | 9 | 14 | 9 | 15 | 8 | | 14,375 | 5,805 |
|  |  |  |  |  |  |  |  |  | |  |  |
| ***n* of reads mapping goat genome** | 11 | 10 | 12 | 0 | 9 | 5 | 17 | 7 | | 8,875 | 5.055 |
| **Total *n* of reads mapping different host genomes** | 55,079 | 76,932 | 21,215 | 80,236 | 8,586 | 4,079 | 32,788 | 15,884 | | 36,849.875 | 30,224.225 |
|  |  |  |  |  |  |  |  |  | |  |  |
| **Total *n* of reads unmapped** | 667,496 | 262,353 | 509,287 | 154,155 | 339,893 | 141,478 | 438,602 | 135,778 | | 331,130.250 | 195,188.457 |
| **Percentage of total reads mapping in different genomes** | 7.623 | 22.675 | 3.999 | 34.232 | 2.464 | 2.802 | 6.956 | 10.473 | |  |  |
| **Percentage of total reads unmapped** | 92.377 | 77.325 | 96.001 | 65.768 | 97.536 | 97.198 | 93.044 | 89.527 | |  |  |

**Supplementary Table 1 B. Paired design of wastewater samples collected before and after treatment across slaughterhouses.** PRE samples correspond to raw influent wastewater, while POST samples correspond to effluent after physicochemical treatment.

| **Sample name** | **Sampling date** | **Treatment stage** |
| --- | --- | --- |
| 1-PRE | 17/10/2023 | Pre-treatment |
| 1-POST | 17/10/2023 | Post-treatment |
| 2-PRE | 04/03/2024 | Pre-treatment |
| 2-POST | 04/03/2024 | Post-treatment |
| 3-PRE | 12/12/2023 | Pre-treatment |
| 3-POST | 12/12/2023 | Post-treatment |
| 4-PRE | 12/02/2024 | Pre-treatment |
| 4-POST | 12/02/2024 | Post-treatment |
