# Supplementary material for: Long-read metagenomics reveals stable resistome and microbiome in treated Italian slaughterhouse wastewater: a preliminary study
Source: Microbiol Spectr. 2026 Jun 22;14(8):e01562-26. doi: 10.1128/spectrum.01562-26 (PMC13435776; doi:10.1128/spectrum.01562-26)
Supplement: Supplementary file 3 [file spectrum.01562-26-s0005.docx]

**Supplementary Table 4A. Pairwise Bray–Curtis dissimilarity values among wastewater samples before and after treatment.**

| **Sample** | | **1-PRE** | | **1-POST** | **2-PRE** | **2-POST** | **3-PRE** | **3-POST** | **4-PRE** | **4-POST** |
| --- | --- | --- | --- | --- | --- | --- | --- | --- | --- | --- |
|  |  |  | |  |  |  |  |  |  |  |
| **1-PRE** |  | - | | 0,020 | 0,676 | 0,379 | 0,545 | 0,505 | 0,517 | 0,503 |
| **1-POST** |  |  | | - | 0,673 | 0,379 | 0,542 | 0,508 | 0,510 | 0,502 |
| **2-PRE** |  |  | |  | - | 0,707 | 0,440 | 0,668 | 0,412 | 0,754 |
| **2-POST** |  |  | |  |  | - | 0,628 | 0,482 | 0,587 | 0,432 |
| **3-PRE** |  |  | |  |  |  | - | 0,516 | 0,297 | 0,636 |
| **3-POST** |  |  | |  |  |  |  | - | 0,559 | 0,376 |
| **4-PRE** |  |  | |  |  |  |  |  | - | 0,642 |
| **4-POST** |  | |  |  |  |  |  |  |  | - |

**Supplementary Table 4B. PERMANOVA based on Bray–Curtis beta diversity comparing PRE and POST wastewater samples.**

| **Factor** | **Df** | **Sum of Squares** | **R²** | **F** | **p-value** |
| --- | --- | --- | --- | --- | --- |
| **Treatment group (PRE vs POST)** | 1 | 0.32762 | 0.32783 | 29.264 | 0.25 |
| **Residuals** | 6 | 0.67172 | 0.67217 | — | — |
| **Total** | 7 | 0.99934 | 1.00000 | — | — |

**Supplementary Table 4C. Beta-dispersion analyses based on Bray–Curtis beta diversity comparing PRE and POST wastewater samples.**

| **Factor** | **Df** | **Sum of Squares** | **Mean of Squares** | **F** | **Permutations** | **p-value** |
| --- | --- | --- | --- | --- | --- | --- |
| Groups | 1 | 0.000267 | 0.0002666 | 0.027 | 999 | 0.91 |
| Residuals | 6 | 0.059146 | 0.0098577 | — | — | — |
